# A Dynamic Autocatalytic Network Model of Therapeutic Change

**DOI:** 10.3390/e24040547

**Published:** 2022-04-13

**Authors:** Kirthana Ganesh, Liane Gabora

**Affiliations:** Fipke Centre for Innovative Research, Department of Psychology, University of British Columbia, 3247 University Way, Kelowna, BC V1V 1V7, Canada

**Keywords:** autocatalytic network, creativity, conceptual network, psychotherapy, therapeutic change, uncertainty, worldview

## Abstract

Psychotherapy involves the modification of a client’s worldview to reduce distress and enhance well-being. We take a human dynamical systems approach to modeling this process, using Reflexively Autocatalytic foodset-derived (RAF) networks. RAFs have been used to model the self-organization of adaptive networks associated with the origin and early evolution of both biological life, as well as the evolution and development of the kind of cognitive structure necessary for cultural evolution. The RAF approach is applicable in these seemingly disparate cases because it provides a theoretical framework for formally describing under what conditions systems composed of elements that interact and ‘catalyze’ the formation of new elements collectively become integrated wholes. In our application, the elements are mental representations, and the whole is a conceptual network. The initial components—referred to as *foodset items*—are mental representations that are innate, or were acquired through social learning or individual learning (of *pre-existing* information). The new elements—referred to as *foodset-derived items*—are mental representations that result from creative thought (resulting in *new* information). In clinical psychology, a client’s distress may be due to, or exacerbated by, one or more beliefs that diminish self-esteem. Such beliefs may be formed and sustained through distorted thinking, and the tendency to interpret ambiguous events as confirmation of these beliefs. We view psychotherapy as a creative collaborative process between therapist and client, in which the output is not an artwork or invention but a more well-adapted worldview and approach to life on the part of the client. In this paper, we model a hypothetical albeit representative example of the formation and dissolution of such beliefs over the course of a therapist–client interaction using RAF networks. We show how the therapist is able to elicit this worldview from the client and create a conceptualization of the client’s concerns. We then formally demonstrate four distinct ways in which the therapist is able to facilitate change in the client’s worldview: (1) challenging the client’s negative interpretations of events, (2) providing direct evidence that runs contrary to and counteracts the client’s distressing beliefs, (3) using self-disclosure to provide examples of strategies one can use to diffuse a negative conclusion, and (4) reinforcing the client’s attempts to assimilate such strategies into their own ways of thinking. We then discuss the implications of such an approach to expanding our knowledge of the development of mental health concerns and the trajectory of the therapeutic change.

## 1. Introduction

While the efficacy of psychotherapy as a form of treatment has been clearly established [1], there is uncertainty about *why* it works [2]. Statistical approaches model the psychotherapeutic process using moderator and mediator variables [3,4], but this does not go far toward explaining each mind’s unique, self-organizing network of associations, how this structure took shape, and how it responds to psychotherapy. We have only a hazy understanding of how specific elements of the psychotherapy process contribute to therapeutic change [5]. This paper aims to take a step forward toward a more precise understanding of what happens in psychotherapy and what makes it effective using a dynamical systems framework to model the interconnected, self-organizing nature of an individual’s worldview, and its dynamical change over time. In so doing, we aim to both strengthen the theoretical bases of psychotherapy, and sharpen our capacity to improve it in practice.

We view psychotherapy as a creative collaborative process between therapist and client, in which the output is not an artwork or invention, but an outlook and approach to life on the part of the client. A client’s outlook and behavior flows from the web of knowledge and experience that collectively constitute a way of seeing the world and being in the world, i.e., a *worldview*. What makes network approaches to cognition particularly promising is that this web of knowledge and experience can be described as a network consisting of loosely connected clusters (i.e., the network has an intermediate degree of modularity) that can be characterized using tools from network science [6,7,8,9,10,11,12]. Thus, the network approach offers a novel way of understanding mental health concerns and their treatment [13]. Psychotherapy (whether it be behavioral, cognitive, etc.) attempts to ‘destabilize’ a distressing or pathological mental state and shift the individual toward a healthier mental state [13,14,15]. What distinguishes the ‘autocatalytic’ approach to cognition taken here from other network-based models of cognition is its capacity to generate new elements (such as mental representations or schemas) out of interactions between existing ones. This makes it useful in the context of describing psychotherapeutic change, though the approach has been applied more broadly to other forms of cognitive change as well [16,17,18]. Since therapy entails change in the structure and dynamics of this network, network science seems a natural place to start in modeling the therapeutic process.

We note that it is not simply the case that positive interpretations (i.e., narratives that make the individual feel good) are adaptive while negative interpretations (i.e., narratives that make the individual feel bad) are maladaptive. It is often necessary that negative situations be acknowledged as such to spur action and find solutions; however, one worldview may predispose the individual to overcoming challenges and finding opportunities, while another leads to unnecessary distress and feelings of helplessness. Thus, the therapist strives to help the client to ‘unravel’ their worldview just enough to ‘reweave’ it into one that is, for that client, adaptive.

One’s society and culture provides stories, narratives, scripts, and schemas, as well as larger conceptual frameworks (such as science or religion) that offer prescriptions for integrating them into a worldview; however, since no one else is privy to an individual’s entire repertoire of personal experiences and intimate observations, the worldview one weaves is ultimately unique. Much as, for any given set of dots there are multiple ways of connecting them, for any given set of experiences or mental representations, there may be many ways of integrating them into a worldview. However, this sentence is not to be interpreted as implying that mental representations are, indeed, anything like ‘dots’; they clearly have a context-dependent inner structure. A worldview may selectively include, or exclude, certain experiences and positively (or negatively) valenced items, (or weight them more strongly). Some worldviews may be more adaptive than others, i.e., more conducive to thriving, personal growth, and the well-being of the individual and their social and environmental milieu.

We model the psychotherapeutic process using a certain kind of network referred to as a Reflexively Autocatalytic foodset-derived (RAF) network. Though the term ‘autocatalytic networks’ reflects their initial application to the origin of life [19,20], RAFs provide a general mathematical setting for studying networks that arose out of earlier work in graph theory [21]. The term *reflexively* is used in its mathematical sense, meaning that each part is related to the whole. The term *autocatalytic* will be defined more precisely shortly, but for now it refers to the fact that the whole can be reconstituted through interactions amongst its parts. The term *foodset* refers to the elements that are initially present, as opposed to those that are the products of interactions amongst them. As in other network science approaches, the nodes of the network represent units of information such as words, concepts, memories, or mental representations of concrete or abstract knowledge, and connections between nodes (by way of free association, shared features, or co-occurrences) are represented as edges (For example, ‘chair’ and ‘wood’ are nodes, and the relationship between them, i.e., wood can be used to create a chair, is represented as an edge).^1^

What differentiates RAFs from other approaches in network science is that the nodes are not just passive transmitters of activation; they actively galvanize, or ‘catalyze’ the synthesis of novel (‘foodset-derived’) nodes from existing ones (the ‘foodset’). The generalized RAF setting is conducive to the development of efficient (i.e., polynomial time), algorithms for questions that are computationally intractable (i.e., NP-hard. [30]). These features make RAFs uniquely suited to model how new structure grows out of earlier structure, i.e., generative network growth [30]. Such generativity may result in phase transitions to a network that is self-sustaining and self-organizing [31,32,33], as well as potentially able to evolve, i.e., exhibit cumulative, adaptive, open-ended change [34,35]. For this reason, RAFs have been used to model the origins of evolutionary processes, both biological—the origin of life (OOL) [36,37]—and cultural—the origin of culture (OOC), or more specifically, the kind of cognitive structure capable of generating cumulative, adaptive, open-ended innovation [17,38,39,40,41]. In a OOL context, RAFs were used to develop the hypothesis that life began as, not as a single self-replicating molecule, but as a set of molecules that, through catalyzed reactions, collectively reconstituted the whole [20]. Autocatalytic network theory has successfully demonstrated—mathematically or using simulations [36,42], and with real biochemical systems [37]—how self-maintaining structures that evolve and replicate can emerge from nonliving molecules. Because RAF nodes modify network structure, the RAF framework is consistent with the goal of understanding not just how networks are structured but also how they dynamically restructure themselves in response to internal and external pressures.

When autocatalytic models are applied in a cognitive context as they are here, they model not just network structure, but how the network reconfigures itself on the fly in response to changing needs and experiences. The observation that, similar to living organisms, cognitive networks are self-sustaining, self-organizing, and self-reproducing [43,44,45,46] suggests that cognitive networks constitute a second level of autocatalytic structure. By *cognitive network,* we refer to an individual’s web of concepts, language terms, and their associations, as well as knowledge and memories, and how they are structured. The self-sustaining nature of a cognitive network is evident in the tendency to reduce cognitive dissonance, resolve inconsistencies, and preserve existing schemas in the face of new information. Although the contents of a cognitive network change over time, it maintains integrity as a relatively coherent whole. Its spontaneously self-organizing nature is evident in the capacity to combine remote associates [47] (such as combining CHOCOLATE and BUNNY to invent CHOCOLATE BUNNY).^2^ The cognitive autocatalytic network replicates in a piecemeal manner through social learning and story-telling. Psychotherapeutic change facilitates the piecemeal replication of adaptive perspectives and habits, as well as the reorganization of relationships between elements of the client’s worldview, and the RAF approach is well-suited to model this.

We begin with an introduction to the psychotherapeutic process. We then introduce RAF networks, and elaborate how they are used in this paper. Next, we present A RAF network model of therapeutic change facilitated by the therapist. The paper closes with implications of the model for fostering a concrete understanding of psychotherapeutic techniques, and suggestions for extending and testing it. A list of abbreviations, and a glossary of terms and their definitions can be found in the Appendix A.

## 2. The Therapeutic Process

Psychotherapy, or ‘talk therapy’ is rooted in formal Western medicine since the late 1800s and practices to alleviate human distress through conversation, known as the ‘moral cure’ has existed formally and informally for centuries [48]. Despite the fact that many effective forms of psychotherapy have been developed, there is uncertainty regarding the mechanisms of therapeutic change [2,5]. Different constructs across therapies show overlap, leading to difficulty with defining their roles and relative importance in the therapeutic process [49]. Thus, a paradigm shift towards dimensional, systemic, and interactional approaches to understanding mental illness and psychotherapy is warranted [50,51,52]. Multi-modal, multi-perspective research methods that enable us to capture the process of therapy in real-time are the future of psychotherapy research [53]. A complex systems approach can thus help re-conceptualize mental health concerns and treatment to more accurately represent the dynamic interactions involved [13], and help us understand therapeutic change [54].

The collaborative nature of psychotherapy is dyadic, and each member of the dyad influences the other through verbal, nonverbal, and physiological synchrony [55,56,57,58]. However, the client and therapist rarely have independently corroborative estimates of important process variables such as the therapeutic alliance [59], and any conceptualization of therapeutic change should make room for both perceptions. RAF networks can accommodate both perspectives within a single framework. The model presented in this paper focuses more on the change in the client’s mind, but the approach has the potential to be expanded to include the therapist, and thereby capture the bidirectional exchange more comprehensively.

Clients generally enter therapy to alleviate distress and increase well-being. Sometimes the decision is prompted by a specific problem, a troubling experience or belief, or something that is difficult to accept. A client may report symptoms of depression, such as sadness, hopelessness, and decreased motivation due to, for example, difficulties with interpersonal relationships. As such, the client’s approach towards such relationships, whether it is the thoughts, emotions, or behaviors involved, are currently insufficient/ineffective in helping them achieve their goals. Therapy may bring about modification of their cognitive network, by enabling them to find a new perspective on a problem or a troubling experience or belief, or come to terms with something they could not accept, thus integrating it into their worldview.

There are significant parallels between the creative process involved in, say, inventing something new, and the process of problem-solving in psychotherapy. Creativity flourishes in situations that involve a tension between uncertainty and constraints [60], or what has been referred to as *enabling constraints* [17,61]. We posit that the forging of a new, healthy, integrated conception of the world and one’s place in it is a creative process, and by cultivating a client-tailored therapeutic interaction, the therapist acts as the midwife of this process.^3^

## 3. Rationale for the Approach

Similar to other cognitive network approaches, RAF networks are hierarchical yet decentralized, and they can be analyzed with respect to density, connectedness, and size. They also draw upon the conventional cognitive science notion of *spreading activation* through nodes of a concept graph, and techniques such as shortest path distance and clustering analysis. However, the RAF approach differs from other network approaches used psychology and cognitive science in a number of respects:

### 3.1. ‘Reactions’ and ‘Catalysis’

A RAF consists of not just nodes connected by edges, but also *reactions,* or interactions that trigger, or ‘catalyze’ new nodes. Thus, RAF nodes are not merely passive recipients of spreading activation; they actively redirect it. For example, seeing a Superman movie might spark a child who believes she is powerless to draw herself as a superhero. In this example, the ‘reactants’ are the mental representation of herself as powerless, as well as her drawing skills. The ‘catalyst’ is the Superman movie, and the ‘product’ is the drawing of herself as a superhero.

In chemistry, and in applications of RAFs to the origin of life, the term ‘reaction’ refers to an interaction between molecules. For consistency, in cognitive applications of RAFs, the term *reaction* is used to refer to an interaction between *mental representations* (MRs) in a cognitive network. It may involve *representational redescription* (RR): the re-coding of information in working memory by modifying, restructuring, elaborating, and/or performing mental operations upon it, or possibly in the absence of an external cue [62]. RR can also involve a shift of perspective, and it can result in a flash of insight, or a newly perceived application for an old idea.^4^ The issue of which concepts participate in a given reaction is discussed and mathematically modeled in [40].

RAFs also have two kinds of edges: reaction edges and catalysis edges. *Reaction edges* are similar to the edges in conventional network science approaches. (They can be thought of as the ‘anatomy’ of the network). *Catalysis edges* are more dynamic. (They can be thought of as the ‘physiology’ of the network). MRs are *catalytic* because they not only participate in certain reactions, but also facilitate—or catalyze—other reactions.^5^ In chemistry, a catalyst speeds up a reaction that would otherwise occur very slowly if at all. By endowing cognitive network models with the capacity for catalysis we can model how one idea or environmental stimulus, triggers a mental operation (such as concept combination, or RR) that would otherwise occur very slowly or not at all. For example, realization of a novel or creative outcome (such as the drawing of a superhero version of oneself) may not have occurred without the galvanizing or ‘catalyzing’ impact of the experience of watching a Superman movie.

As in chemistry, the cognitive equivalent of a ‘catalyzed reaction’ may trigger another reaction, and so forth, resulting in a *reaction sequence.* In cognitive models, this reaction sequence is a stream of thought, which may ultimately have arisen from a problem, question, or cognitive dissonance. For example, the ultimate source of the cognitive reaction sequence culminating in the creation of a superhero character may be the desire never to feel powerless.

The rationale for treating mental representations (MRs) as catalysts comes, in part, from the literature on concepts, which provides extensive evidence that when concepts act as contexts for each other, their meanings change [66,67]. For example, an ISLAND has the property ‘surrounded by water’, but (hopefully) not a KITCHEN ISLAND. KITCHEN momentarily reconfigures the cognitive network, altering the perceived meaning of ISLAND. Such alterations in meaning are often nontrivial, and defy classical logic [68]; however, quantum models of concept interactions provided a means of formalizing the process by which a context (such as the goal of creating a spot to cut food) spontaneously bridges remote associates (such as KITCHEN and ISLAND) [23,24,25]. Although cognitive RAF models are influenced by how context is modeled in these quantum models of concepts, it is not committed to any formal approach to modeling context. Context is considered to be anything in the external environment, or anything from long-term memory that influences how a MR is instantiated in working memory. The extent to which one MR modifies the meaning of another is referred to as its *reactivity*.

In sum, the RAF approach incorporates not just cognitive change due to adjustments in association strengths, but also cognitive change due to the prompting or ‘catalysis’ of new nodes. The resulting network is dynamic both in terms of structure (e.g., new nodes can be generated), and information flow (e.g., newly generated nodes can result in new information pathways).

### 3.2. Foodset versus Foodset-Derived

Another key feature of RAF models is the distinction between *foodset* items, which came into existence *outside* the network in question, and *foodset-derived* items, which come about through ‘catalyzed reactions’ *within* the network in question. An individual’s ‘mental foodset’, or simply, *foodset* includes memories of direct experiences, i.e., that came about by way of the senses, including any knowledge that has come about through individual learning (of pre-existing information) by way of direct experience in the world, or through social learning processes such as imitation or classroom learning. The foodset may also include innate responses, such as the fear of heights and corresponding inclination to back away from a precipice. Together, these innate responses, direct experiences, and socially transmitted knowledge constitute the *raw materials* from which the individual’s cognitive network is built. Thus, the worldview is “grounded in perception” because it grows out from this foodset.

Much as bricks and bags of mortar do not constitute a house, the foodset does not constitute a mental model of the world, a worldview. The set of *foodset-derived* items consists of mental contents that were generated by that individual from scratch (and constitute new information) using foodset elements, or perhaps other foodset-derived elements, as ingredients. The generation of foodset-derived items occurs by way of mental operations such as problem solving, insight, deduction, induction, and abduction. Since the elements of the worldview described by foodset-derived items are not grounded in perception, they can be viewed as ‘useful fictions’. Thus, if the therapist responds to powerlessness in a certain way, and the client learns and (later) copies that response, that way of responding is an item in the client’s foodset; however, if the therapist acts as a midwife for the client’s expression of emotions associated with powerlessness during a therapy session, the artwork is a foodset-derived item^6^. The approach thereby distinguishes between conceptual shifts originating within the mind of a given individual, and those that originated by others, and were learnt or assimilated by that individual. What foodset items all have in common is that they are raw materials the individual has at his/her disposal to work with in the generation of new MRs, and this generation of new MRs is a key component of to the conceptual change that occurs during psychotherapy.

In cognitive networks, the distinction between foodset and foodset-derived provides a natural means of grounding abstract concepts in direct experiences; foodset-derived elements emerge through ‘reactions,’ that can be traced back to foodset items. This enables us to identify the necessary precursor ideas ideas for the emergence of new understandings, and the mental operations a given individual carried out to generate a particular idea. This capacity to model the reconfiguration of a cognitive network makes RAFs ideal for the study of change that occurs in psychotherapy.

### 3.3. Generational Cognitive/Cultural Change

Because of the distinction between foodset and foodset-derived MRs it is possible to tag new insights with their point of origin (i.e., keep track of whose mind did each idea arose in), and track cumulative change step by step within and across individuals. We posit that a mind can be described in terms of nested and overlapping RAFs, and these RAFs are what evolve through culture. Thus, each human lifetime constitutes a small segment of our collective cultural evolutionary lineage (see [34,38,39]). Each generation builds on the accomplishments of the previous generation, such that items that were foodset-derived for one generation become elements of the foodset for the next, and this kind of cumulative cultural evolution has also been modeled, both computationally [69,70,71,72], and mathematically using RAFs [17,34,38,39,40]. For example, an early hominid invented the first tool by realizing that repeatedly striking one stone with another can produce a stone that is sharp, and the mental script of how to make this tool is described as a foodset-derived item in that individual’s mind. This mental script was shared with peers, who in turn transmitted it to others, and in their minds it was a foodset item. As a more psychological example, ‘flattery makes friends’ constituted a foodset-derived item in the mind of the first person to have this thought. He or she may have shared this notion with others, and in their mind it is a foodset item, but one of them may build on it by realizing that imitation can be flattering, and therefore a route to friendship, in which case this new version is a foodset-derived idea (i.e., ‘imitation is the sincerest form of flattery’). Thus, our worldviews consist largely of information that has already been preprocessed into scripts, schemas, stories, and narratives by previous generations, and such ‘chunks’ constrain the shape of one’s worldview.

### 3.4. Potential to Scale Up

In this initial application of RAF networks to the therapeutic process, the examples used are fairly simple; however, a significant strength of the approach is that RAFs can scale up. The RAF approach can be used to analyze and detect phase transitions in extremely complex networks (such as the phase transition from no-RAF to RAF in Kauffman’s [20] binary polymer model) that have proven intractable using other analytic approaches [37,73].

## 4. Reflextively Autocatalytic Foodset-Derived Networks (RAFs)

Let us now define the term *Reflexively Autocatalytic and foodset-derived network* (RAF) more precisely [30,31,32,35,74]. The term *reflexive* is used in its mathematical sense to mean that each component is related (directly or indirectly) to the whole. As mentioned in Section 1, the term *autocatalytic* refers to the fact that the whole can be reconstituted through interactions amongst its components. A network qualifies as A RAF network if it meets the following two criteria:

(1) It is *reflexively autocatalytic*: each reaction $r\in R^{\prime }$ is catalyzed by at least one element type that is either produced by $R^{\prime }$ or is present in the foodset *F*. This is sometimes referred to as *closure*.
(2) It is *F-generated*: all reactants in $R^{\prime }$ can be generated from the foodset *F* by using a series of reactions only from $R^{\prime }$ itself.

Thus, an RAF is a non-empty subset $R^{\prime }\subseteq R$ of reactions that meets these two criteria: it is reflexively autocatalytic, and F-generated.

The term *catalytic reaction system* refers to a network consisting of components that can catalyze the generation of other components, and a catalytic reaction system can consist of one or more RAFs. The largest RAF, which subsumes all other RAFs, is referred to as the *maxRAF*. All other RAFs are referred to as *subRAFs*. A RAF that cannot be broken down into smaller RAFs is referred to as an irreducible RAFs, or *irrRAF*. It is not necessarily the case that a catalytic reaction system contains a RAF, but if it does contain one or more RAFs, it has a unique maxRAF. To put this more formally, if the network contains a RAF, then the collection of all its RAFs forms a partially ordered set (i.e., a poset) under set inclusion, with the maxRAF as its unique maximal element. RAFs can evolve, as demonstrated both mathematically and in simulation studies, through selective proliferation and drift acting on possible subRAFs of the maxRAF [32,75].

The catalytic reaction system is a tuple Q=(X,R,C,F) consisting of a set *X* of types, a set R of reactions, a catalysis set *C* indicating which molecule types catalyze which reactions, and a subset *F* of *X* referred to as the foodset. A subset $R^{\prime }$ of the full reaction set R of a catalytic reaction system Q forms a RAF if is both *collectively autocatalytic* (by the first criterion, because each of its reactions is catalyzed by some component in the system), and *self-sustaining* (because of the *F*-generated criterion).

RAFs can enlarge and combine. The union of any two (or more) subRAFs forms a RAF (which explains why there is a unique maximal RAF). These two subRAFs may be disjoint, or they may have some reactions in common. A subRAF $R^{\prime }$ can also expand by combining with a ‘co-RAF’, where a *co-RAF* is any nonempty set of reactions that is not A RAF but, when combined with $R^{\prime }$, forms A RAF. RAF expansion can also be extrinsically driven. For example, it can be due to social learning of a new story or skill, i.e., a change in the foodset. External stimuli may even trigger a ‘reaction’; for example, the instruction to ‘think creatively’ may ‘catalyze’ the generation of new ideas. In a therapeutic context, this could take the form of of a question or suggestion, such as to try seeing a particular interpersonal situation from the other person’s perspective.

RAFs emerge in a system of interacting components when their complexity passes a critical threshold [20,33]. In applications of RAF networks to model the origin of life, the components are polymers: molecules made up of repeated units called monomers. In applications of RAF networks to model cognitive networks, the components are MRs. The RAF framework provides a means of analyzing the emergence of complex networks, identifying how phase transitions might occur, and at what parameter values. The phase transition from no RAF to A RAF has been analyzed (mathematically and through simulations), and applied to biochemical [31,32,33,36,42], cognitive [38,39,40], and ecological [76], systems.

During childhood, the individual assimilates experiences, stories, narratives, scripts, and schemas, and gradually weaves them into a network of understandings, and this process has been analyzed using the RAF framework [16]. Eventually these pieces of knowledge condense into a maxRAF, which grows and changes through childhood and beyond. Once the maxRAF encompasses the majority of these fragments of knowledge they are mutually accessible. At this point, the child no longer requires a cue or reminder in the environment to access something from memory because the maxRAF provides a route from any one idea to any other. The maxRAF enables the individual to make plans and predictions, generate metaphors, and adapt old techniques or ideas to new circumstances; however, while an integrated maxRAF network helps the individual think creatively and effectively negotiate the environment, it may be conducive to distorted thinking, and other biases that are *emotionally* dysfunctional, and result in mental health concerns.

An individual’s worldviews could be said to be self-contained in that there exists a maxRAF—meta-RAF of sorts—that encompasses the majority of the individual’s subRAFs. We have modeled not just how this maxRAF forms over the course of child development [16], but how the capacity for such a maxRAF evolved over the course of human history [34,38,39,40]. The worldviews of different individuals are interconnected in that and subRAFs of one individual are mirrored in subRAFs of another, and indeed, RAF structure can ‘flow’ and extend across individuals [17,18].

### Cognitive RAFs

Whern RAFs are used to model cognition, all MRs in a given individual *i* are denoted $X_{i}$, and a specific MR $x=x_{i}$ in $X_{i}$ is denoted by writing $x\in X_{i}$. MRs are either *foodset MRs*, or *foodset-derived MRs*. The *foodset* of individual *i*, denoted $F_{i}$, encompasses MRs that are either innately present, or that are the result of direct experience in the world, whether it be by way of social learning, or by way of natural or artificial stimuli. Thus, $F_{i}$ has multiple components:

- $S_{i}$ denotes the set of MRs arising through direct experience that have been encoded in individual *i*’s memory. It includes:
  - MRs obtained through social learning from the communication of an MR $x_{j}$ by another individual *j*, denoted $S_{i}\left[x_{j}\right]$.
  - MRs obtained through individual learning, denoted $S_{i}\left[\ell \right]$.
- Any *innate knowledge* with which individual *i* is born, denoted $I_{i}$.

$F_{i}$ includes information obtained through social interaction with *someone else* who acquired this knowledge as a result of their own creative or analytical thought processes. (For example, if individual *i* learns from individual *j* that it is ok to say no, this is an instance of social learning, and “it’s ok to say no” becomes a member of $F_{i}$. In contrast, if individual *i* realizes on their own that it is ok to say no, then “it’s ok to say no” is not a member of $F_{i}$.) $F_{i}$ includes everything in individual *i*’s long-term memory that did not result from individual *i* engaging in RR. $F_{i}$ also includes pre-existing information obtained by *i* through individual learning (which, as stated earlier, involves learning from the environment by non-social means), so long as this information retains the form in which it was originally perceived (and does not undergo redescription or restructuring through abstract thought). The crucial distinction between foodset and non-foodset items is not whether another person was involved, nor whether the MR was originally obtained through abstract thought (by *someone*), but whether the abstract thought process originated in the mind of the individual *i* in question.

Foodset-derived elements are denoted $\neg F_{i}$. Thus, $\neg F_{i}$ refers to mental contents that are *not* part of $F_{i}$ (i.e. $\neg F_{i}$ consists of all the products b∈B of all reactions $r\in R_{i}$). In particular, $\neg F_{i}$ includes the products of any reactions derived from $F_{i}$ and encoded in individual *i*’s memory. Its contents come about through mental operations *by the individual in question* on the foodset; in other words, foodset-derived items are the direct product of RR. Thus, $\neg F_{i}$ includes everything in long-term memory that *was* the result of one’s own thought processes. $\neg F_{i}$ may include a MR in which social learning played a role, so long as the most recent modification to this MR was a catalytic event (i.e., it involved RR).

A single instance of RR in individual *i* is referred to as a *reaction,* and denoted $r\in R_{i}$. RR is often applied recursively, such that the output of one thought serves as the input to the next. The set of reactions that can be catalyzed by a given MR *x* in individual *i* is denoted $C_{i}\left[x\right]$. The entire set of MRs either *undergoing* or *resulting from* *r* is denoted *A* or *B*, respectively, and a member of the set of MRs undergoing or resulting from reaction *r* is denoted a∈A or b∈B. Thus, for example, if a client has the idea of expressing her grief at the passing of her father by painting a scene in which the clouds evoke her deceased father, the concepts FATHER and CLOUD are reactants in *A*, and the resulting concept FATHER-CLOUD is a product in *B*. This conceptual shift, treated as a ‘reaction’, is ‘catalyzed’ by the client’s desire to process the death of her father. It is in this way that the RAF approach tags novelty (in this case, the painting) with its point of origin (by showing in which mind in a cultural lineage it was a foodset-derived item).

The set of *all* possible reactions in individual *i* is denoted $R_{i}$. The mental contents of the mind, including all MRs and all RR events, is denoted $X_{i}⊕R_{i}$. Recall that the set of all MRs in individual *i*, including both the food set and the food set-derived items, is denoted $X_{i}$. $R_{i}$ and $C_{i}$ are not prescribed up front; because $C_{i}$ includes remindings and associations on the basis of one or more shared features, different kinds of interactions are possible between any given pair of MRs. Nonetheless, it makes sense mathematically to refer to $R_{i}$ and $C_{i}$ as sets.

## 5. Model

We now apply RAF theory to the modification of a client’s worldview in psychotherapy. To make this more concrete, we explain our model using a hypothetical interaction between a fictional yet representative therapist named Thera, and a client, named Clive. We show how the therapist elicits adaptive change in a dysfunctional belief in the client’s worldview.

### 5.1. Intake Form and Thera’s Emerging Mental Model of Clive

Thera learns from an intake form that Clive is a young man with no strong friendships, who has experienced debilitating social anxiety for years. His decision to start therapy was prompted by a recent incident in which his wife called him a ‘moron’ during an intense disagreement. This, in conjunction with several other earlier incidents, have forced him to conclude that he is ‘stupid’.

**Figure 1:**
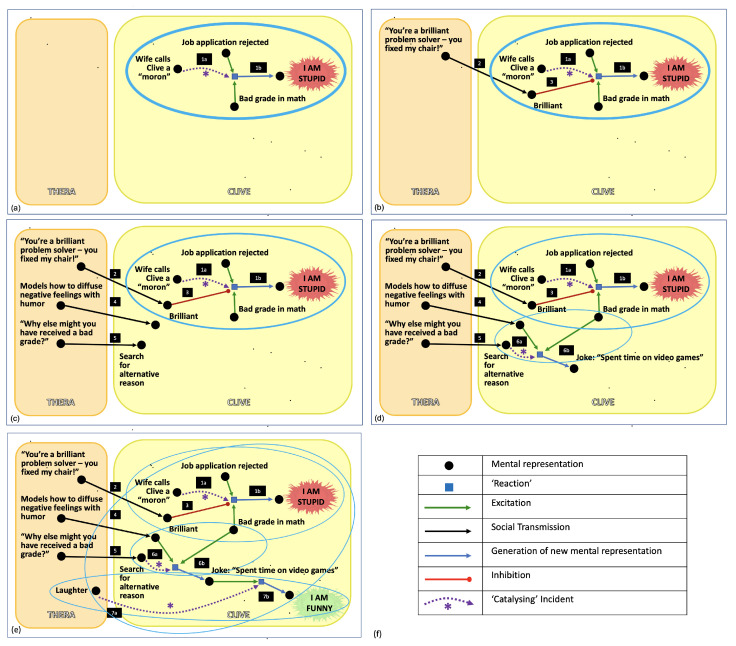
(**a**) RAF model of how client’s worldview is altered over the course of a psychotherapy session. Initially, Thera’s conception of Clive consists solely of what she read on his intake form. Following a ‘catalyzing incident’ in which his wife called him a “moron”, he been interpreting other events as confirmation of the distressing belief, ‘I am stupid’. Collectively, these elements constitute a stable RAF, as indicated by the thick blue line forming an oval around them. The thickness of this line indicates that the RAF has a large impact on Clive’s thinking. (**b**) Thera praises Clive’s brilliant problem solving ability, which generates a new foodset item, the notion that he is ‘brilliant’. Since this is inconsistent with the belief ‘I am stupid’, it reduces the impact of that RAF, as indicated by the fact that the width of the line forming a blue oval is now thinner. (**c**) Two more foodset items are socially transmitted from Thera to Clive. (**d**) Making use of what Thera modeled for Clive about diffusing negative feelings using humor, he makes a joke. The joke is catalyzed by Thera’s prompt to explore alternate explanations for why he received a bad grade. The joke depletes negative feelings associated with the bad grade, such that it is less able to serve as a ‘reactant’ to support the belief that he is unintelligent, as illustrated by the further dissolution of the oval representing that RAF. His joke constitutes a second RAF. (**e**) Thera’s laughter at Clive’s joke catalyzes a new belief, ‘I am funny’, which enhances his self-esteem, and forms a third RAF. These first three RAFs, which are irr-RAFs because they cannot be reduced further, interact with one another, and together form a maxRAF, which encompasses them all. (**f**) A key describing the symbols used in the various panels.

A portion of Thera’s mental model of her client after reading this report is shown in the first panel (panel (a)) of Figure 1. For the relationship between Thera and Clive to develop, they need to establish some form of psychological contact [77]. Thera welcomes Clive to the room and sits down. As she introduces herself, her body posture is relaxed. She provides several forms of non-verbal encouragement, such as smiling, nodding, supportive interjections, and eye contact. This makes him feel like someone worthy of the attention of another, which lifts his self-confidence, and allows him to speak more comfortably.

As psychotherapy proceeds, Thera starts to unearth information about Clive’s worldview using therapeutic techniques such as reflections, clarifications, and open-ended questions. Clive shares other significant life experiences that affected his self-esteem, such as getting a D- in a high school math class, and not getting a job after working hard to prepare for the interview. By asking questions such as, “Why do you think you received a poor grade?”, and “How did that make you feel?”, Thera is able to gather information regarding Clive’s interpretations of these events. From this, using her preexisting knowledge about social anxiety and mental health, she extrapolates Clive’s concerns, and builds a mental model of him in her mind.

While Clive’s belief that he is unintelligent is based in real-life adverse experiences—modeled here as ‘foodset items’—it involves extrapolation, and possibly distortion. It appears that the triggering incident in which his wife called him a moron served as a ‘catalyzing incident’ that initiated a tendency toward confirmation bias, such that he reinterprets other past and present events as confirmation of the belief, ‘I am stupid’. This, in turn, is damaging his self-esteem. The confirmation bias has thus exacerbated his preexisting concerns; his anxiety in social situations is now more severe due to his belief that he is ‘stupid’, and he has become isolated and lonely.

We can view what is happening here from the perspective of Clive’s worldview as a whole. The self-organizing, self-mending nature of a worldview can create a system of internal feedback combined with external influences that can sustain and amplify an existing (and in this case, negative) narrative [78]. Clive has come to interpret interactions with others as consistent with his negative self-image, and his mistrust and withdrawal have created a positive feedback system that reinforces his belief about social interactions.

To model this using the RAF approach and thereby understand it in more precise terms, the client, Clive, is denoted *C*, the poor grade is denoted $G_{C}$, and not getting the position he applied for is denoted $P_{C}$. These memories (and likely others) serve as the raw materials, or reactants, for Clive’s confirmation bias. First we note that they are part of his foodset, as follows:

(1) $$G_{C},P_{C}\in F_{C}.$$

$G_{C}$ and $P_{C}$ become reinterpreted as evidence for the belief “I am stupid”, denoted $b1_{C}$. The catalyzing event that initiates this, i.e., the fight with his wife where she called Clive a ‘moron’, is denoted by $m_{C}$. Thus, this process is described as follows:

(2) $$G_{C}+P_{C}\overset{m_{c}}{\to }b1_{C}\in \neg F_{C},\neg F_{C}\mapsto \neg F_{C}\cup \left\{b1_{C}\right\}.$$

The catalysis of $G_{C}$ and $P_{C}$ by $m_{C}$ is **Step 1a** in Panel (a) of Figure 1. The resulting formation of $b1_{C}$ is **Step 1b** in Panel (a) of Figure 1. The ¬ sign indicates that $b1_{C}$ is not part of the foodset, i.e., it is a foodset-derived item. The portion of Equation (2) after the comma simply tells us that the set of foodset-derived items has expanded to include $b1_{C}$.

Note that this little cognitive network now satisfies the conditions for A RAF; (1) all reactions (in this case, there is just r1) can proceed, because the needed catalyst is present, and (2) the needed reactants (MRs of events that could be interpreted as confirmation of his lack of intelligence) are also present. The greater the extent to which ambiguous experiences are interpreted as evidence for the foregone conclusion that he is stupid, the greater to which the MR ‘I am stupid’ constitutes a stable attractor state. (For discussion of attractor states in psychology, see [14,79]). This attractor causes Clive emotional distress, and has an adverse impact on his quality of life.

We now show how Thera extrapolates from what Clive says to build a mental model of him in her mind. Thera interprets Clive’s statement as implying something more general about how he views the world and his place in the world: specifically, that his self-concept is increasingly dominated by the belief that he is (as his wife put it), “a moron”. We use the subscript *T* to refer to Thera. Her foodset, denoted $F_{T}$, includes knowledge of psychopathology and the treatment options available, as well as her growing knowledge of Clive, $C_{T}$. The formation of this knowledge from Clive’s discussion of himself, denoted $C_{C}$, is described as follows:

(3) $$F_{T}\mapsto F_{T}\cup \left\{C_{T}\right\},C_{T}\in S_{T}\left[C_{C}\right].$$

This equation tells us that her foodset now ‘maps to’ a foodset that includes knowledge of Clive, and the part after the comma tells us that knowledge of Clive was socially transmitted from Clive himself.

As Thera’s understanding of Clive’s concerns increases, she feels more emotionally connected to him, which in turn impacts the quality of her responses to him. Clive feels increasingly heard, and begins trusting her. Her reflections, statements, and questions may more readily facilitate adaptive change in Clive that may not have occurred otherwise. Thera’s interactions create perturbations in Clive’ s cognitive network that disrupt the ‘I am stupid’ attractor state described above.

Elaborating on the ‘Clive and Thera’ example, we now show how the RAF model brings to light four distinct ways in which a therapist such as Thera facilitates therapeutic changes in the worldview of a client such as Clive.

### 5.2. Providing Counter-Evidence to Distressing Belief

During the session, Thera’s chair malfunctions. Clive is able to fix the problem by adjusting the various knobs and gears on the chair. Thera says, “You’re a brilliant problem solver—you fixed my chair!” This provides counter-evidence to the belief that he is unintelligent. Her observation, which lifts his mood, temporarily decreases his distress, and enhances his self-concept, is socially transmitted to him, resulting in the new MR: ‘Someone thinks I’m brilliant’. Transmission of the information that he is brilliant, denoted $B_{C}$, is described by Equation (4), as follows:

(4) $$F_{C}\mapsto F_{C}\cup \left\{B_{C}\right\},\mathrm{where}B_{C}\in S_{C}\left(B_{T}\right)$$

Thera’s observation becomes part of Clive’s ‘mental foodset’ because it did not come into existence within Clive’s mind; it was ‘born’ in Thera’s mind, and socially transmitted from Thera to Clive. His self-concept now contains both the constellation of experiences and negative beliefs about his intelligence, described as A RAF, consisting of multiple mutually consistent memories that support his belief that he is ‘stupid’, as well as a new experience that is inconsistent with this RAF. This experience of being described as ‘brilliant’ by Thera therefore has an *inhibitory* role on that RAF; it weakens the strength of that reaction, thereby diminishing the proclivity to interpret ambiguous events as confirmation of the belief, “I am stupid”. This is depicted in Panel (b) of Figure 1, where **Step 2** refers to the social transmission of words of praise from Thera to Clive, and **Step 3** refers to its inhibitory role on the existing RAF.

### 5.3. Modeling Adaptive Mindset through Self-Disclosure

Thera models how to consciously resist the tendency to interpret ambiguous evidence in a negative manner through the use of self-disclosure regarding how she manages self-critical thoughts in her own life. She also spontaneously models adaptive responses in her interactions with him. She bumps her elbow on the desk, and then laughs at her clumsiness. The laughter enables her to re-frame the thought ‘I am clumsy’ into something innocuous. Thera’s social transmission of this to him is **Step 4** in Panel (c) of Figure 1. Where the socially transmitted laughter is denoted $L_{C}$, this process is described as follows:

(5) $$F_{C}\mapsto F_{C}\cup \left\{L_{C}\right\},\mathrm{where}L_{C}\in S_{C}\left(L_{T}\right)$$

The possibility of responding to one’s inadequacies with laughter and/or self- deprecating humor is a new and striking concept for Clive. As such, this experience forms a foodset element in Clive’s mind. His decision to use this strategy himself (i.e., to incorporate it as a reactant in subsequent steps) depends on a number of variables, such as the degree to which he trusts and respects the therapist, his motivation to change, and so forth. (Elaboration of these variables is beyond the scope of this discussion).

### 5.4. Catalyzing Alternative Explanations

Thera not only transmits *existing* knowledge that Clive can import, wholesale, into his worldview, she also prompts the independent formation of *new* information, specifically tailored to his personality, that are conducive to adaptive perspectives and behaviors, and help him resolve or come to terms with the issues he faces. This could take the form of asking Clive questions that prompt him to reconsider existing beliefs, or by challenging Clive’s beliefs directly. For example, Thera might as him, “Might there be other reasons that you didn’t do so well on that math test?” This question gently challenges Clive’s forgone interpretation of the event. It prompts him to explore reasons other than that he is unintelligent. This is depicted in panel (c) of Figure 1, as **Step 5**.

Clive responds, laughing, “Well yeah, I spent a lot of time playing video games”. This alternative interpretation does not play into the notion that Clive is unintelligent, and it shows that he has assimilated her proclivity to diffuse a negative conclusion with humor. It generates a *new* interpretation. This is depicted in Panel d of Figure 1, where **Step 6a** shows the catalyzing event (i.e., the search for an alternate explanation), and **Step 6b** shows the product of this ‘reaction’, Clive’s joke.

We describe this in terms of RAFs as follows. Clive’s memory of getting a bad grade on the test, denoted $G_{C}$, undergoes change, so it serves as a reactant that transforms through RR to the product, the joke about video games, denoted $V_{C}$. This new interpretation is provoked, or ‘catalyzed’ by Thera’s question, denoted $q_{T}$. We describe this as follows:

(6) $$G_{C}\overset{q_{T}}{\to }V_{C}\in \neg F_{C},\neg F_{C}\mapsto \neg F_{C}\cup \left\{V_{C}\right\}.$$

This conceptual shift transforms one or more element(s) of the foodset $F_{C}$ into a new foodset-derived MR, $V_{C}$, i.e., a member of $\neg F_{C}$. Clive’s self-concept now contains a new MR—the joke he made—represented as a new node in his cognitive network.

### 5.5. Catalyzing a New Belief That Is Adaptive

When Clive dilutes the potency of this once-distressing memory with humor, Thera laughs. This laughter, denoted as $l_{T}$ catalyzes a new belief in Clive’s mind, ‘I am funny’, denoted as $b2_{C}$. This new belief buoys his self-esteem, and reduces his distress. The joke, denoted as $J_{C}$ serves as a reactant. Thus, the reaction is described as follows:

(7) $$J_{C}\overset{l_{T}}{\to }b2_{C}\in \neg F_{C},\neg F_{C}\mapsto \neg F_{C}\cup \left\{J_{C}\right\}.$$

This is depicted in Panel (e) of Figure 1, where **Step 7a** refers to the catalyzing event, i.e., Thera’s laughter, and **Step 7b** refers to the resulting formation of the new belief, ‘I am funny’.

Using these four distinct methods, Thera simultaneously reduces the strength of a distressing belief that was damaging his self-concept, and facilitate the creation of a new belief that enhances Clive’s self-concept, as illustrated by the relative thickness of the RAFs in Figure 1.

We note there are now hierarchical levels of RAF structure, composed of interacting RAFs that collectively form a maxRAF (panel (e) of Figure 1) (The nodes in the therapist’s mind associated with Steps 2, 4, and 5 in Figure 1 can either be included in this maxRAF or not, since from the RAF point of view they are merely copies of the corresponding elements in the client’s worldview). We note also that RAF structure extends across the two individuals, providing a means of formally describing the dyadic relationship between therapist and client, and the emergence of a therapeutic alliance between them.

## 6. Discussion and Conclusions

We presented a RAF model of how a therapist fosters self-esteem and well-being in the client. The model illustrated four distinct ways by which a therapist accomplishes this: (1) providing direct examples/evidence contrary to a client’s distressing belief about themselves, (2) challenging the client’s existing interpretations of events, (3) using self-disclosure, provide examples of strategies for diffusing the potency of a negative belief, (4) reinforcing the client’s attempts to assimilate such strategies in their own ways of thinking.

As discussed in Section 3 and Section 4, RAF networks have been used to model the origins of evolutionary processes, biological (the origin of life) as well as cultural (the origin of cumulative innovation). We think this is not coincidental; indeed, elsewhere, we showed that both the evolution of early life and cultural evolution are instantiations of a primitive form of evolution—i.e., cumulative, adaptive, open-ended change—referred to as Self-Other Reorganization (SOR) [34,80,81]. Instead of replication using a self-assembly code, SOR entails internal self-organizing and self-maintaining processes within entities, as well as interaction between entities. The argument for SOR bolsters the argument that they share a deep structure, and thus strengthens the rationale for applying RAFs in both domains. In any case, the RAF approach to modeling therapeutic change is consistent with the theory that humans possess two levels of complex, adaptive, self-organizing structure: an organismal level, and a psychological level [43,45,82]. Psychological research tends to be data rich and theory poor [83], and psychological theorizing remains fragmented [84]. Psychotherapy research relies on momentary snapshots of the perceptions of client and therapist; it is vague about the nature of psychotherapeutic change, i.e., what happens at the level of mental representations and their interrelations and interactions, and how this kind of micro-level change alters the global structure of the client’s worldview [5]. We take a first step towards such a global understanding in this paper. We posit that psychotherapeutic processes affect people not just at the individual level but at the society level, by providing a means to the creative transformation and cultural evolution of human worldviews.

Traditional methods for studying psychotherapeutic change have limitations [54] that the complex systems approach is well positioned to overcome [85,86,87,88], by enabling psychotherapy to be modeled and understood more precisely, using tools that embed it in a larger framework that includes other systems and disciplines. The above model of the therapeutic process provides a framework for empirical data collection and analysis. A next step is to incorporate into such a model specific factors that affect therapeutic outcomes (such as the degree of trust in the therapist). The impact of the therapeutic alliance between therapist and client on the therapy outcome is well-known [54,89]. It would be interesting to analyze psychotherapy sessions to track cognitive change, and the emergence of a therapeutic alliance, and its impact on this change. Our model accommodates the perspectives of both the therapist and client. While we have chosen to emphasize the client in this interaction, the RAF approach can also model potential changes in the therapist’s worldview. The RAF approach could also be used to investigate a number of other issues related to psychopathology and treatment, such as the development of mental illness, the trajectory of various mental health concerns, and whether there are differences in the conceptual frameworks of individuals experiencing depression and those with anxiety. It could also be used to model the impact of different types of psychotherapy on conceptual network structure, and the impact of this structure on mental health and well-being. One promising possibility is to study whether individual differences in reliance on foodset versus foodset-derived information sources (i.e., the propensity to think things through for oneself) culminate in different kinds of conceptual networks, which might differentially affeact therapeutic progress. In addition, using RAF networks to precisely model the psychotherapeutic process could be informative in the design and execution of computerized psychotherapies [90,91], or as an aid to the human psychotherapist for keeping track of, and visually depicting, specific interactions in the psychotherapy process and their outcome. We are a long way from this, but in keeping with the adage “a picture is worth a thousand words”, the RAF framework for psychotherapy could form the basis for a software program that enables the therapist to visualize and identify change in RAF structure as it occurs over the course of psychotherapy, and to visualize desired possible future states of their clients’ worldviews.

The RAF approach offers an established mathematical framework for integrating research on creative cognition, semantic networks, and the kinds of structures that exhibit cumulative, adaptive, open-ended change, i.e., that evolve, with a similarly dynamic process of psychotherapy. Though still in its infancy, it has the potential to provide a new way of understanding how the therapeutic alliance works, one that embeds psychotherapy research in the formal study of self-organizing structures and their role in evolutionary processes.

## Footnotes

1 As explained elsewhere [16], the ‘autocatalytic’ approach taken here is consistent with distributed models of mental representations in memory [22], and with quantum models of their interactions [23,24,25,26,27,28,29].

2 This proposed cognitive level of autocatalytic structure is not merely an extension of organismal needs; indeed, the biological and cognitive/cultural levels of endogenous control can be at odds (e.g., a scientist immersed in solving a problem may neglect offspring, or forget to eat).

3 Many describe the generative aspect of cognition as a ‘birthing’ of new ideas or attitudes, and this word directly captures the therapist’s role in facilitating this process.

4 Creative insights often arise subconsciously from just beyond the bounds of working memory [63].

5 The use of the word ‘catalyze’ in a cognitive context extends beyond autocatalytic models of cognition [64,65], though these other approaches are purely descriptive.

6 This distinction is not as black and white as portrayed here, but for simplicity, we do not address that subtlety for now.

## Abbreviations

The following abbreviations are used in this manuscript:

CRS	Catalytic reaction system
RAF	Reflexively Autocatalytic foodset-derived network
MR	Mental representation
RR	Representational redescription
SOC	Self-organized criticality

## Appendix A. Glossary of Terms

**Abstract thought**: the processing of internally sourced mental contents.
**Autocatalytic**: the whole can be reconstituted through interactions amongst its parts.
**Catalyst**: facilitates a transition that would otherwise be highly unlikely to occur. Here, the role of catalyst is played by a problem, desire, or need, or a realization or external stimuli that trigger a thought that would be highly unlikely to occur otherwise. For example, if a stranger on the street reminds you of a deceased relative, and this triggers a memory of being with that person, the strange (or more precisely, your mental representation of the stranger plays the role of a catalyst.)
**Catalytic reaction system**: a network of interrelated parts, such as a conceptual network.
**Closed RAF**: A RAF that is stable unless the foodset changes or the reactions they take part in changes. (Formally, a closed RAF is A RAF that contains every reaction in the network that has each of its reactants and at least one catalyst present either in the foodset or as a product of some reaction in the RAF.) The maxRAF is always closed. The *closure* of any subRAF will contain the original subRAF, and be larger (unless the original subRAF was already closed).
**Conceptual network**: a web of shared properties, contexts, associations, and relationships of logic, causation, and so forth, that bind them together.
**Co-RAF**: a nonempty set of reactions that is not A RAF on its own, but that forms A RAF when combined with an existing set of reactions.
**Catalytic reaction system**: a network of components, such as a network of catalytic molecules, or a conceptual network.
**Foodset, *F***: the elements that are initially present, as opposed to those that are the products of interactions amongst them.
**foodset-derived (sometimes called F-generated, or foodset-derived), ¬F**: an element that can be generated from the foodset *F* through a series of reactions in $R^{\prime }$ itself. That is, an element of the network that is *not* part of the foodset. The term ‘foodset-derived’ is more often used in the cognitive application of RAFs.
**Individual learning**: obtaining pre-existing information from the environment by nonsocial means through direct perception.
**IrrRAF**: A RAF that is irreducible, i.e., cannot be broken down into smaller RAFs.
**MaxRAF**: the largest RAF in the network. It includes all other RAFs.
**Mental representation (MR)**. Items in declarative or procedural memory composed of one or more concepts or percepts, and which came about through individual learning, social learning, or abstract thought. The set of all mental representations in individual *i* is denoted $X_{i}$. (As mentioned in the text, we emphasize that although we use the terms ‘mental representation’, we are sympathetic with the view that what we call mental representations do not ‘represent’, but act as contextually elicited bridges between the mind and the world.)
**Reflexive**: each part is related to the whole.
**Reflexively autocatalytic**: each reaction $r\in R^{\prime }$ is catalyzed by at least one element type that is either produced by $R^{\prime }$ or is present in the foodset *F*.
**Phase transition**: rapid transition from one state to another.
**Reactant**: a mental representation that participates in a given ‘reaction’, i.e., an event that alters the structure of the conceptual network.
**Reaction**: a change of state or interaction between existing elements that results in a new element. The set of all possible reactions in individual *i* is denoted $R_{i}$.
**Representational redescription (RR)**: conceptual restructuring that causes a mental representation to change. In the RAF framework this is modeled as a reaction.
**Self-organized criticality (SOC)**: a phenomenon wherein, through simple local interactions, complex systems tend to find a critical state poised at the cusp of a transition between order and chaos, from which a single small perturbation occasionally exerts a disproportionately large effect.
**SubRAF**: A RAF that is not the maxRAF. It is a component of the maxRAF.
**Transient RAF**: a subRAF that is not closed. A transient RAF may add additional reactions until it becomes closed.
